# High prevalence of methotrexate intolerance in rheumatoid arthritis patients: a cross-sectional study

**DOI:** 10.1186/s41927-025-00466-2

**Published:** 2025-07-21

**Authors:** Harjit Singh Nalwa, Tushar Singh Barwal, Parul Chugh, Neha Singh, Neeraj Jain, Lalit Duggal, N. K. Ganguly, Ved Chaturvedi, Shivani Arora Mittal

**Affiliations:** 1 Department of Biotechnology and Research, Sir Ganga Ram Hospital, New Delhi, 110060 India; 2 Department of Rheumatology and Clinical Immunology, Sir Ganga Ram Hospital, New Delhi, 110060 India

**Keywords:** Rheumatoid arthritis, Methotrexate, Questionnaire, Intolerance, Methotrexate intolerance severity score (MISS), DAS-28

## Abstract

**Background:**

Methotrexate (MTX) is the most commonly used disease-modifying antirheumatic drug (DMARD) for treating rheumatoid arthritis (RA). However, MTX use is associated with gastrointestinal adverse effects in a number of patients. Early detection of MTX intolerance could help modify the treatment strategy, thereby ensuring patient compliance and response. In the present study we aimed to identify the prevalence of MTX intolerance, and associated risk factors in a cohort of Indian RA patients receiving oral MTX therapy.

**Methods:**

In this cross- sectional study, RA patients who were in regular use of oral or subcutaneous MTX for a minimum duration of three months were included. The participants were evaluated based on their responses to the methotrexate intolerance severity score (MISS) questionnaire. Patients with a MISS score ≥ 6 were considered MTX intolerant. Demographic data encompassing the patient’s age, sex, diet, MTX dosage, duration of use, route of administration, other medication, and disease activity assessed using the DAS-28 CRP was collected using a standardized patient history sheet.

**Results:**

Out of 200 adult RA patients, 86% were females with an average age of 49.25 ± 11.89 years, and the average duration of MTX use was 46.16 ± 53.40 months. A high prevalence of MTX intolerance (34.5%) was observed in RA patients. Nausea (85.5%) followed by abdominal discomfort (59.42%) were the most prevalent symptoms in intolerant patients. Furthermore, using multivariate analysis, we observed a positive association of MTX intolerance with female gender, disease severity, and MTX dose.

**Conclusion:**

Although MTX is the one of the most commonly used medication for the treatment of RA, there is significant intolerance to this drug among adult RA patients. The symptoms observed not only occur after MTX intake but are also present before intake (anticipatory) and while thinking of taking MTX (associative). Our data indicates that a MTX dose of 15 mg/week or greater may be associated with intolerance. There is a need to objectively monitor RA patients to identify MTX intolerance early on to ensure mitigation steps for effective treatment response.

## Background

Rheumatoid Arthritis (RA) is an autoimmune disorder inducing severe inflammation of joints, which may cause irreversible bone and cartilage damage, often leading to disability [1]. Based on the global incidence data, the age-standardized prevalence rate for RA increased from 207.46 per 1,00,000 in 1990 to 224.25 per 1,00,000 in 2019 and is projected to increase by a rate of 18.23 and 8.34 per 1,00,000 people for females and males, respectively, by the year 2030 [2]. Consequently, RA imposes significant individual and social burden, with the individual burden stemming from musculoskeletal impairments, reducing the quality of life and diminishing physical function. In contrast, socioeconomic burden includes significant direct medical costs, decreased work capacity and reduced social participation [3]. The treatment for RA involves the use of disease-modifying antirheumatic drugs (DMARDs), and methotrexate (MTX) is the drug of choice due to its low cost, high efficacy and acceptable safety profile [4, 5]. Mechanistically, MTX is an anti-folate that inhibits DNA synthesis and exhibits immunomodulatory action in rapidly proliferating inflammatory cells. Despite being one of the most widely used DMARD, MTX has some side effects [6]. MTX acts by increasing adenosine concentration in and outside cells. Given the ubiquitous presence of adenosine receptors throughout the body, especially in the central nervous system (CNS), some gastrointestinal symptoms could be associative and anticipatory. Additionally, several behavioral symptoms are also identified with MTX intake. Hence, based on the above symptoms, the methotrexate intolerance severity score (MISS) questionnaire has been validated to identify MTX intolerance in RA patients [7]. This questionnaire scores the gastrointestinal (before/after/and even upon thinking of MTX) and behavioral side effects. Using this questionnaire, a cumulative MISS score of 6 or more points with at least 1 point in the anticipatory, associative or behavioral symptom category is defined as MTX intolerance. This questionnaire has been used to identify the prevalence of MTX intolerance in different countries [8–10].

MTX intolerance is likely to develop within the first year of treatment, although its onset may vary considerably depending on individual factors and ethnic differences [11]. Additionally, genetics [12] and dietary factors, such as folic acid intake, can also affect its prevalence. For instance, genetic variations in the enzymes responsible for MTX metabolism can lead to intolerance [13]. Identification of intolerant patients early on is key to modify the treatment in a timely manner and improve patient compliance [14]. There is limited literature on the prevalence of MTX intolerance and its correlating factors from India [15]. The present study was designed to assess the prevalence of MTX intolerance and its associated risk factors using the MISS questionnaire in a cohort of RA patients from a tertiary care center in India. In developing countries such as India, most RA patients are prescribed oral MTX therapy as the preferred drug of choice. It is therefore imperative to study the frequency and development of intolerance to MTX among RA patients in the Indian population. Early detection of MTX intolerance could help modify the treatment strategy, thereby ensuring patient compliance and response.

## Materials and methods

### Study design and approval

This cross- sectional analytical study was conducted in the Department of Rheumatology and Clinical Immunology, and the Department of Biotechnology and Research, Sir Ganga Ram Hospital, New Delhi, India. The patients were assessed from the tenure of October 2020 to October 2023. The study was conducted after receiving approval from the institutional ethics committee (SGRH EC NO: - EC/10/20/1796). All patients provided their written informed consent prior to their inclusion in the study.

### Patient eligibility and assessment

**Fig. 1:**
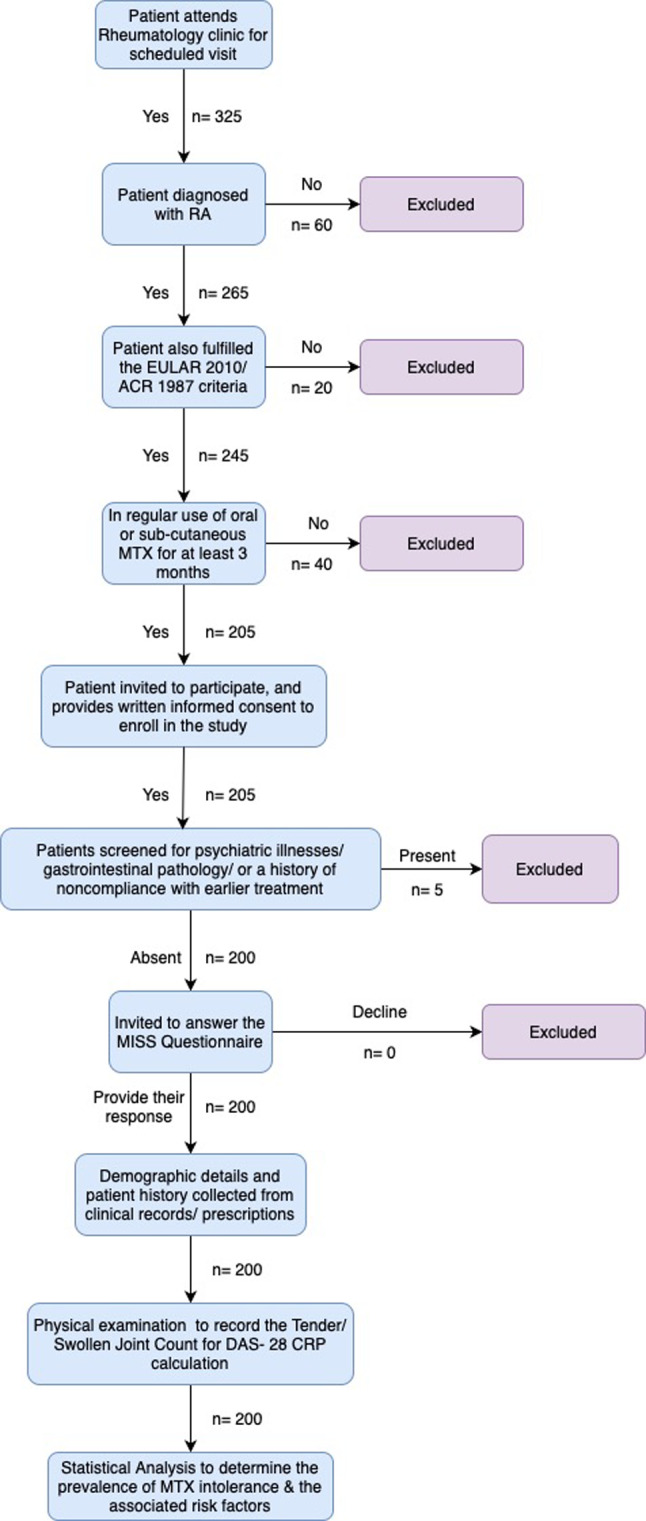
Flow diagram illustrating the process of patient inclusion

Patients diagnosed with RA, and also fulfilling the European League Against Rheumatism (EULAR) 2010 criteria/American College of Rheumatology (ACR) 1987 criteria [16, 17] and who were in regular use of oral or sub-cutaneous route MTX for at least 3 months were invited to answer the MISS questionnaire. We employed a cross- sectional design, with data collected in real-time from patients attending the rheumatology outpatient clinic. Patient enrollment was done for confirmed adult RA cases when they presented to the clinic for their routine visit. Illiterate patients were interviewed by one of the investigators, who ensured that each patient had a clear understanding of all items. Additionally, all the patients were interviewed by a single investigator to ensure uniformity of data collection. Patients with psychiatric illnesses, gastrointestinal pathology, or a history of noncompliance with earlier treatment were excluded from the study. We defined non- compliance as any patient who missed 3 consecutive doses of medication, and did not adhere to treatment for 3 consecutive weeks. The validated MISS questionnaire consists of four domains, including abdominal pain (three questions), nausea (three questions), vomiting (two questions) and behavioral symptoms (four questions) [18]. The symptoms taken into consideration include symptoms after MTX administration, before MTX intake (anticipatory symptoms) and when thinking of MTX (associative symptoms). The investigator scored the possible symptoms for each complaint, wherein (0) score was associated with no complaint, (1) with mild, (2) with moderate, and (3) with severe complaint. Patients with a collated score of 6 or more with at least 1 point in the anticipatory, associative or behavioral symptom category were considered to be intolerant. Furthermore, demographic data, including the patient’s age, sex, diet (vegetarian or non- vegetarian), MTX dosage, duration of MTX use, route of administration, concomitant medications, and disease activity as assessed by the Disease Activity Score in 28 joints (DAS28-CRP), was collected from clinical records and prescriptions, and recorded on a standardized patient history sheet. In addition, the small joints of the patient’s hands were examined to note the presence of any kind of joint deformity, defined as distortion and disfigurement resulting in swelling, pain, or loss of joint function. A flow diagram illustrating the process and framework of patient inclusion has been depicted in Fig. 1.

### Statistical analysis

SPSS Version 28.0 (Released 2021; IBM SPSS Statistics for Windows, Version 28.0. Armonk, NY: IBM Corp) was used for statistical analysis. The study began by looking at the association between MTX intolerance and both demographic and clinical characteristics, using Pearson’s Chi-Square for categorical data and t-tests or Mann-Whitney tests for quantitative data, depending on their distribution. A univariate analysis was conducted to evaluate the association of variables such as age, sex, corticosteroid use, joint deformity, and MTX dosage. Symptoms such as stomach ache, nausea, vomiting, and behavioral symptoms were analyzed collectively, irrespective of their classification as after, anticipatory, or associative symptoms. Use of other DMARD drugs was not included in the analysis, as they were used by less than 10% of the patients. Factors with a potential relationship with MTX intolerance (p-value < 0.05) using univariate analysis were further examined using multivariate logistic regression to identify independent risk factors. The multivariate logistic regression was conducted using a Forward Likelihood Ratio (LR) model. Odds ratios (OR) were calculated to predict the likelihood of MTX intolerance. The model’s success was determined by a p-value < 0.05, which defined statistical significance. The continuous variables in the study were reported as Mean ± Standard Deviation (SD). Additionally, the association of MTX dose with intolerance was determined using an ROC curve.

## Results

### Demographic details

Two hundred confirmed RA patients who met the selection criteria of MTX duration of at least 3 months were included in the study. Table 1 represents the demographic details of these patients, wherein the majority were females (86%) with a mean age of 49.25 ± 11.89 years, and an average duration of MTX therapy of 46.16 ± 53.40 months. The mean DAS28-CRP score was 4.98 ± 1.23. As most patients recruited in our study were those with long standing RA, 76% of the patients were receiving a combination of MTX and hydroxychloroquine, whereas a small subset of patients were treated with a JAK inhibitor in conjunction with MTX (7.5%). Notably, only one patient was administered a TNF inhibitor in combination with MTX. It is noteworthy that as biologicals are a more expensive option and as we excluded patients without MTX therapy, we had very few patients in our cohort receiving this modality.

**Table 1:** Patients’ baseline characteristics at the time of completing the MISS questionnaire

Characteristics		Number (Percentage)
Number of Patients		200
Gender	Male	28 (14%)
	Female	172 (86%)
Mean Age (Years)		49.25 ± 11.89
Route of Administration	Oral	199 (99.50%)
	Subcutaneous	1 (0.50%)
MTX Dose	Up to 10 mg/week	25 (12.50%)
	15 mg/week	151 (75.50%)
	20 mg/week	24 (12%)
Rheumatoid Factor	Positive	71 (35.50%)
	Negative	18 (9%)
	Unknown	111 (55.50%)
Duration of MTX therapy (Months)	Up to and including 12 months	72 (36%)
	Between 12 to 48 Months	71 (35.50%)
	More than 48 Months	57 (28.50%)
Mean Duration of MTX therapy (Months)		46.16 ± 53.40
Mean TJC		12.3 ± 6.26
Mean SJC		4.31 ± 5.09
Mean CRP		5.36 ± 9.19
Mean Patient Global Health score		48.77 ± 25.41
Mean DAS- 28 score		4.98 ± 1.23
Glucocorticoids		62 (31%)
Folic Acid		194 (97%)
Antacids		9 (4.50%)
Biologics| Targeted Synthetic DMARDS	Janus kinase (JAK) inhibitor (Tofacitinib)	15 (7.50%)
	TNF blocker	1 (0.50%)
NSAIDs & other Analgesics (*N* = 60)	Etoricoxib	27 (13.50%)
	Tramadol + Paracetamol	8 (4%)
	Pregabalin	3 (1.50%)
	Gabapentin	2 (1%)
	Naproxen	12 (6%)
	Others	8 (4%)
Glucocorticoids (*N* = 62)	Prednisolone	44 (22%)
	Deflazacort	13 (6.50%)
	Others	5 (2.50%)
Supplements (*N* = 91)	Calcium & Vitamin D_3_ (Shelcal)	58 (29%)
	Curcuminoids (Carcuviva)	6 (3%)
	Glucosamine + Collagen	3 (1.50%)
	Others	24 (12%)
DMARDs (*N* = 384)	MTX	200 (100%)
	Hydroxychloroquine	152 (76%)
	Sulfasalazine	16 (8%)
	Leflunomide	15 (7.50%)
	Others	1 (0.50%)

### Methotrexate intolerance: prevalence and symptoms

Based on the MISS scoring, MTX intolerance was observed in 34.5% of RA patients. The most frequent symptoms observed in intolerant patients were nausea (85.5%), followed by stomach ache (59.42%) and vomiting (43.47%). Notably, a majority of these symptoms were observed after taking MTX. However, for the nausea category, even the anticipatory (55%) and associative symptoms (69.6%) were highly prevalent. Furthermore, the behavioral symptom of restlessness was also identified with significant frequency (43.47%). The distribution of symptoms in patients with and without MTX intolerance is detailed in Table 2.

**Table 2:** Prevalence of MTX-related symptoms in RA patients, as assessed using the MISS questionnaire

Symptom	Symptom	Response	Total (*n* = 200)	Tolerant (*n* = 131)	Intolerant (*n* = 69)
Stomach Ache	Stomach Ache - After	No Complaint	138 (69.0%)	110 (84.0%)	28 (40.6%)
		Mild	20 (10.0%)	12 (9.2%)	8 (11.6%)
		Moderate	17 (8.5%)	5 (3.8%)	12 (17.4%)
		Severe	25 (12.5%)	4 (3.1%)	21 (30.4%)
	Stomach Ache - Anticipatory	No Complaint	182 (91.0%)	128 (97.7%)	54 (78.3%)
		Mild	8 (4.0%)	2 (1.5%)	6 (8.7%)
		Moderate	5 (2.5%)	1 (0.8%)	4 (5.8%)
		Severe	5 (2.5%)	0 (0.0%)	5 (7.2%)
	Stomach Ache - Associative	No Complaint	184 (92.0%)	129 (98.5%)	55 (79.7%)
		Mild	7 (3.5%)	2 (1.5%)	5 (7.2%)
		Moderate	5 (2.5%)	0 (0.0%)	5 (7.2%)
		Severe	4 (2.0%)	0 (0.0%)	4 (5.8%)
Nausea	Nausea - After	No Complaint	113 (56.5%)	103 (78.6%)	10 (14.5%)
		Mild	29 (14.5%)	14 (10.7%)	15 (21.7%)
		Moderate	22 (11.0%)	10 (7.6%)	12 (17.4%)
		Severe	36 (18.0%)	4 (3.1%)	32 (46.4%)
	Nausea - Anticipatory	No Complaint	152 (76.0%)	121 (92.4%)	31 (44.9%)
		Mild	31 (15.5%)	8 (6.1%)	23 (33.3%)
		Moderate	9 (4.5%)	2 (1.5%)	7 (10.1%)
		Severe	8 (4.0%)	0 (0.0%)	8 (11.6%)
	Nausea - Associative	No Complaint	130 (65.0%)	109 (83.2%)	21 (30.4%)
		Mild	38 (19.0%)	19 (14.5%)	19 (27.5%)
		Moderate	20 (10.0%)	3 (2.3%)	17 (24.6%)
		Severe	12 (6.0%)	0 (0.0%)	12 (17.4%)
Vomiting	Vomiting - After	No Complaint	164 (82.0%)	125 (95.4%)	39 (56.5%)
		Mild	11 (5.5%)	2 (1.5%)	9 (13.0%)
		Moderate	10 (5.0%)	2 (1.5%)	8 (11.6%)
		Severe	15 (7.5%)	2 (1.5%)	13 (18.8%)
	Vomiting - Anticipatory	No Complaint	188 (94.0%)	129 (98.5%)	59 (85.5%)
		Mild	9 (4.5%)	1 (0.8%)	8 (11.6%)
		Moderate	3 (1.5%)	1 (0.8%)	2 (2.9%)
Behavioral	Behavioral - Restlessness	No Complaint	154 (77.0%)	115 (87.8%)	39 (56.5%)
		Mild	27 (13.5%)	13 (9.9%)	14 (20.3%)
		Moderate	7 (3.5%)	0 (0.0%)	7 (10.1%)
		Severe	12 (6.0%)	3 (2.3%)	9 (13.0%)
	Behavioral - Crying	Present	4 (2.0%)	0 (0.0%)	4 (5.8%)
		Absent	196 (98.0%)	131 (100.0%)	65 (94.2%)
	Behavioral - Irritability	No Complaint	178 (89.0%)	120 (91.6%)	58 (84.1%)
		Mild	19 (9.5%)	10 (7.6%)	9 (13.0%)
		Moderate	3 (1.5%)	1 (0.8%)	2 (2.9%)
	Behavioral - Refusal of MTX	No Complaint	173 (86.5%)	124 (94.7%)	49 (71.0%)
		Mild	18 (9.0%)	6 (4.6%)	12 (17.4%)
		Moderate	5 (2.5%)	1 (0.8%)	4 (5.8%)
		Severe	4 (2.0%)	0 (0.0%)	4 (5.8%)

### Risk factors associated with MTX intolerance

Most of the RA patients included in this study were in the moderate or severe disease category according to the DAS-28 scoring. MTX-tolerant and intolerant patients had a similar distribution of disease activity. However, the intolerant category had a higher percentage of severe cases (55.07%) than the tolerant category (41.98%). The intolerant cases were found significantly associated with DAS-28 scores using the chi square test (*p*< 0.05), as depicted in Table 3.

**Table 3:** Association between DAS-28 CRP score and methotrexate intolerance

DAS-28 CRP Score	Intolerant Percentage	Tolerant Percentage
Remission (Less than 2.6)	0	2.29%
Mild (2.6 to 3.2)	1.44%	6.87%
Moderate (3.2 to 5.1)	43.47%	48.09%
Severe (More than 5.1)	55.07%	41.98%

All the variables were investigated to find their association with MTX intolerance through univariate and multivariate statistical analysis. Amongst all the drugs, besides hydroxychloroquine, none of the other DMARDs were being used by more than 10% of the patients and hence were not considered for analysis. In univariate analysis, female gender (*p* = 0.046), corticosteroid use (*p* < 0.001), MTX-associated symptoms like stomach ache (*p* < 0.001), nausea (*p* < 0.001), vomiting (*p* < 0.001), behavioral symptoms (*p* < 0.001), joint deformity (*p *= 0.005) and MTX dosage (*p* < 0.001) were found as risk factors associated with MTX intolerance (Table 4). No significant association of MTX intolerance with other variables such as diet or folic acid supplementation were identified.

**Table 4:** Risk factors identified to be significantly associated with MTX intolerance using Univariate analysis

Feature		Total (*n* = 200)	Tolerant (*n* = 131)	Intolerant (*n* = 69)	*p* value
Sex	Female	172 (86.0%)	108 (82.4%)	64 (92.8%)	0.046*
	Male	28 (14.0%)	23 (17.6%)	5 (7.2%)	
Joint Deformity	Present	4 (2.0%)	0 (0.0%)	4 (5.8%)	0.005*
Corticosteroid use	Yes	62 (31.0%)	28 (21.4%)	34 (49.3%)	< 0.001**
MTX Dosage	Mean ± SD	14.96 ± 2.35	14.45 ± 2.04	15.72 ± 2.88	< 0.001**
	Median (IQR)	15 (15–15)	15 (15–15)	15 (15-17.5)	
	Min - Max	8–20	8–20	10–20	

**Table 5:** Risk factors significantly associated with MTX intolerance using Univariate and Multivariate analysis

Variables	Univariate			Multivariate		
	OR	95% CI	*p* value	OR	95% CI	*p* value
Sex (Female)	2.726	0.988–7.524	0.046*	6.311	1.168–34.099	0.032*
Corticosteroid use (Yes)	3.573	1.903–6.711	< 0.001**			
Stomach Ache	2.3	1.730–3.059	< 0.001**	1.912	1.324–2.761	< 0.001**
Nausea	2.537	1.981–3.250	< 0.001**	2.205	1.646–2.954	< 0.001**
Vomiting	2.89	1.922–4.347	< 0.001**	2.106	1.275–3.478	< 0.001**
Behavioral	2.264	1.686–3.040	< 0.001**			
MTX Dosage	1.274	1.106–1.467	< 0.001**	1.341	1.082–1.662	0.007*

OR: Odds ratio; CI: Confidence Interval

Statistically significant *p*-values < 0.05 are indicated by ‘*’, and *p*-values <0.001 are indicated by ‘**’, respectively

**Fig. 2:**
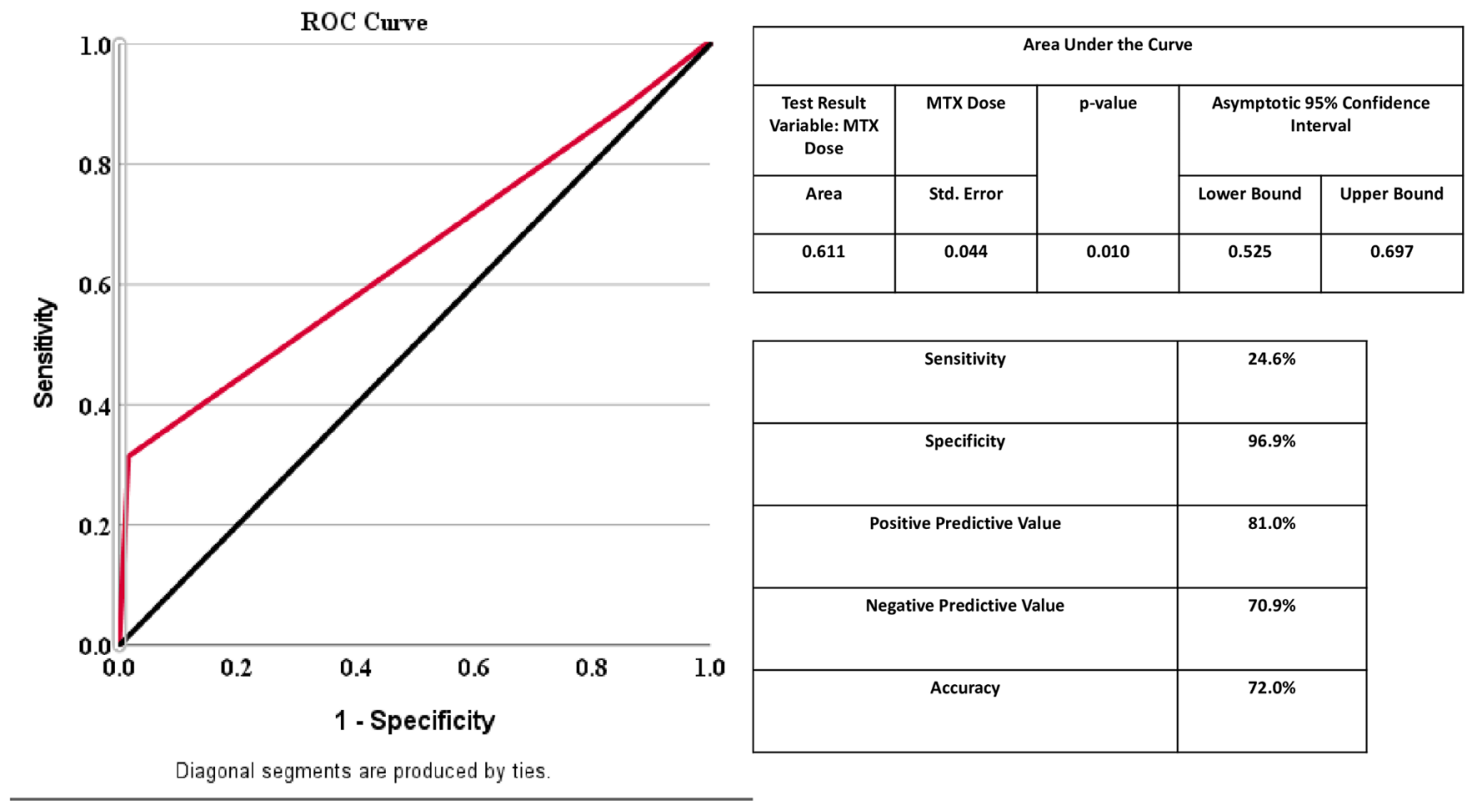
Predictive association of MTX dose with MTX intolerance using the ROC method [ROC: Receiver-Operating characteristic curve]

These associations were further confirmed using multivariate analysis (*p* < 0.05). Using this, female gender, MTX related symptoms, and MTX dosage were retained in the final multivariate regression model, as depicted in Table 5. In multivariate analysis, female gender [OR = 6.311; CI = 1.168–34.099; *p* = 0.032] and MTX dosage [OR = 1.341; CI = 1.082–1.662; *p* = 0.007] were positively associated with MTX intolerance. Furthermore, MTX-associated side effects such as nausea [OR = 2.205; CI = 1.646–2.954; *p* < 0.001], stomach ache [OR = 1.912; CI = 1.324–2.761; *p* < 0.001], and vomiting [OR = 2.106; CI = 1.275–3.478; *p* < 0.001] were also strongly associated with intolerance. Additionally, MTX dosage of 15 mg/week or greater was predictive of intolerance. The predictive ability of MTX intolerance with MTX dose was observed using the ROC curve wherein MTX dose greater than or equal to 15 mg/week was found to be highly associated with MTX intolerance with sensitivity (24.6%), specificity (96.9%), and accuracy (72.0%) as depicted in Fig. 2.

## Discussion

MTX intolerance in RA patients is prevalent across various geographical locations. Despite its efficacy, approximately one-third of patients experience gastrointestinal symptoms during treatment [19]. We have similarly observed a high prevalence of intolerance of 34.5% in adult RA patients, with disease duration ranging from 3 months to more than 48 months. This frequency of intolerance is similar to reports from other countries [20–22]. Conversely, instances of prevalence of low intolerance (11%) were also reported, as indicated by Calasan et al. (2013), underscoring the global challenge of varied intolerance rates across varied geographic and ethnic landscapes [9].

In our study, the primary symptoms reported by the intolerant group were nausea and stomach ache. Additionally, vomiting and behavioral symptoms such as irritability also presented with higher frequencies. Intriguingly, nausea emerged as the predominant symptom not only after MTX administration but also as an anticipatory and associative symptom, corroborating with earlier observations by Amaral et al. (2020) and Sandhu et al. (2020) [10, 12]. Conversely, Fatimah et al. (2016) and Almalag et al. (2020) identified behavioral symptoms as the most recurrent [21, 22], whereas Kanda et al. (2024) identified gastric discomfort as the most common gastrointestinal side effect. The frequency of side effects observed in intolerant RA patients may thus vary in different population groups.

Our findings further highlight the positive correlation between MTX intolerance and drug dosage, corticosteroid use, female gender, and disease severity. Notably, Almalag et al. (2020) reported a significant association between intolerance and behavioral aspects, using the pain score and patient global assessment scale analysis (perceived by the patients) [22]. However, our study did not find the behavioral component as the primary symptom using the symptoms listed in the questionnaire. For the first time, we have reported that a MTX dosage of 15 mg/week or greater is associated with intolerance. In addition, our study included a significant number of male patients (14%) when compared to previous studies.

This study has some limitations. Firstly, there were limited patients with mild disease activity status, and due to the unavailability of RF and ACPA values for all patients, their potential association could not be evaluated. Secondly, as MTX dosage of 15 mg/week is a standard of care for RA patients, our data included very few cases that received higher than this dose. Notably, despite this, MTX dose of 15 mg/week or more was found to be predictive of intolerance with very high specificity. Nonetheless, the role of some confounding variables, such as use of other drugs, NSAIDs and hydroxychloroquine cannot be ruled out. Further confirmation in a cohort of patients receiving variable MTX doses is required. Thirdly, the tool used to assess intolerance was the MISS questionnaire, which is validated and cited widely for use in juvenile and adult RA patients. After the initiation of our study, another questionnaire, MISA, was developed and validated for MTX intolerance in adult RA patients [23]. However, as multiple studies have used MISS as the gold standard and as MISA questionnaire accounts to a lesser degree for associative and anticipatory symptoms, we have used the MISS tool for evaluation. Additionally, we would like to emphasize that the findings of this study are applicable only to a population that primarily receives oral MTX.

Given the substantial patient segment exhibiting intolerance despite proactive folic acid supplementation, this study underscores the pressing need for strategies to mitigate MTX intolerance. Daly et al. have highlighted the efficacy of caffeine synchronization with MTX intake, which may help relieve intolerance [24]. Specifically, a shift from oral to parenteral MTX in patients who develop intolerance within the first year is also found beneficial [25]. Another study conducted by Kromann et al. (2015) also supports that switching from oral to subcutaneous MTX alleviates gastrointestinal adverse effects [26]. Studies on JIA patients demonstrated that EMDR (eye movement desensitization and reprocessing) and pharmacological conditioning may help relieve MTX intolerance [27]. Also, counselling may help relieve the behavioral symptoms associated with intolerance. Thus, there is a need to objectively identify MTX-intolerant patients in clinical settings to facilitate a transition to alternative treatments thereby enhancing patient adherence. Given that gastrointestinal intolerance is well-documented to be higher with the oral route of administration, it would be valuable to include a control group of patients receiving MTX via the subcutaneous route in future studies. Further, more extensive multicentric studies are also required to identify the mechanism, predisposing factors, and mitigation strategies for MTX intolerance in RA patients.

## Conclusion

MTX intolerance occurs frequently among adult RA patients. Our study found a high prevalence of MTX intolerance (34.5%). Nausea (85.5%) followed by abdominal discomfort (59.42%) were the most prevalent symptoms in intolerant patients. Furthermore, our study highlights a positive association of MTX intolerance with female gender, disease severity and MTX dose. Our data indicates that a MTX dose of 15 mg/week or greater seemingly increases the risk of intolerance. There is a need to objectively monitor RA patients in clinics to identify MTX intolerance early on thereby enabling intervention strategies such as change of drug route, folic acid supplementation, use of antiemetics, caffeine supplementation or behavioral therapy, for effective treatment response.

## Data Availability

The datasets used and/or analysed during the current study is provided within the manuscript and further details can be requested from the first author/corresponding author.

## Abbreviations

MTX: Methotrexate
DMARD: Disease-Modifying Antirheumatic Drug
RA: Rheumatoid Arthritis
MISS: Methotrexate Intolerance Severity Score
